# Dynamic Actin Gene Family Evolution in Primates

**DOI:** 10.1155/2013/630803

**Published:** 2013-06-06

**Authors:** Liucun Zhu, Ying Zhang, Yijun Hu, Tieqiao Wen, Qiang Wang

**Affiliations:** ^1^Institute of System Biology, Shanghai University, Shanghai 200444, China; ^2^Yangzhou Breeding Biological Agriculture Technology Co. Ltd., Yangzhou 225200, China; ^3^School of Life Sciences, Shanghai University, Shanghai 200444, China; ^4^State Key Laboratory of Pharmaceutical Biotechnology, School of Life Sciences, Nanjing University, Nanjing 210093, China

## Abstract

Actin is one of the most highly conserved proteins and plays crucial roles in many vital cellular functions. In most eukaryotes, it is encoded by a multigene family. Although the actin gene family has been studied a lot, few investigators focus on the comparison of actin gene family in relative species. Here, the purpose of our study is to systematically investigate characteristics and evolutionary pattern of actin gene family in primates. We identified 233 actin genes in human, chimpanzee, gorilla, orangutan, gibbon, rhesus monkey, and marmoset genomes. Phylogenetic analysis showed that actin genes in the seven species could be divided into two major types of clades: orthologous group versus complex group. Codon usages and gene expression patterns of actin gene copies were highly consistent among the groups because of basic functions needed by the organisms, but much diverged within species due to functional diversification. Besides, many great potential pseudogenes were found with incomplete open reading frames due to frameshifts or early stop codons. These results implied that actin gene family in primates went through “birth and death” model of evolution process. Under this model, actin genes experienced strong negative selection and increased the functional complexity by reproducing themselves.

## 1. Introduction

Actin is an abundant and highly conserved protein that is found in all eukaryotic cells [1]. It is also a major component of total amount of proteins in various kinds of cells [2, 3] and plays an essential role in a variety of important cellular processes including vesicle and organelle movements [4, 5], cell motility [6], cell division [7] and cytokinesis [8], muscle contraction [9], and the establishment and maintenance of cell junctions and cell shape [10]. Except for conventional actin, eukaryotic cells also contain actin-like (ALPs) and actin-related proteins (ARPs), which have well-characterized roles in cytoskeletal functions [11, 12]. Actins, ALPs, and ARPs, comprising a large family of homologous proteins, share the same structural architecture, known as the “actin fold” [13]. These three kinds of proteins are encoded by a multigene family in all animals, plants, and many protozoans examined to date, making up actin superfamily [14–16], which is called actin gene family in this work.

Compared to its functional studies, the organization and evolution of actin gene family are not discussed extensively. Comparisons of nucleotide sequences from the protein coding regions and exon-intron arrangements of related genes provide a means of tracing their evolution pathways [17, 18]. Before the advent of the era of large-scale sequencing, actin gene family has been investigated in many organisms [19–24]. Those results indicate that actin gene family is highly conserved, and the number of actin genes among these organisms is variable. With the development of sequencing technology, recent studies of dynamic actin gene evolution in lower organisms like algae reveal distinct phylogenetic structures and evolution histories [25, 26]. In most of algae, actin genes morphologically cluster with each other on the phylogenetic tree among different algal lineages [25]. In each algal clade of actin tree, at least two subclades are found, in which one contains highly conserved sequences, whereas the other one has very diverged actin isoforms. On the other hand, phylogenetic analysis in dinoflagellates exhibits at least three types of clusters [26]. The first type contains recently duplicated copies within each species, and the other two types form clades including sequences from different species, in which one type contains very similar copies and the other one has divergent copies across species. 

Although there are many studies for this family, no systematic research has been made in primates. Consequently, the purpose of this study is to investigate characteristics and evolutionary pattern of all actin genes in primates. We first identified 233 actin genes including actin-like and actin-related gene plus 337 pseudogenes residing in human, chimpanzee, gorilla, orangutan, gibbon, rhesus monkey, and marmoset genomes. And then, we analyzed and compared their phylogenetic distribution, codon usage, and expression pattern between orthologs and paralogs. Our results indicated that actin genes in primates extraordinarily diverged among paralogs, but were highly conserved across orthologs. In this case, we suggested that actin gene family experienced a duplication followed by mutation process, according with birth and death model of evolution. 

## 2. Material and Method

### 2.1. Identification of Actin Genes

The genome and protein sequences of human (*Homo sapiens*), chimpanzee (*Pan troglodytes*), gorilla (*Gorilla gorilla*), orangutan (*Pongo pygmaeus abelii*), gibbon (*Nomascus leucogenys*), rhesus monkey (*Macaca mulatta*), and marmoset (*Callithrix jacchus*) were downloaded from Ensembl ftp site (ftp://ftp.ensembl.org/pub/release-69/fasta/). We identified actin genes as follows: first of all, we downloaded protein sequences which were limited to genes with actin domain (Pfam: PF00022) from Biomart [27] (website: http://asia.ensembl.org/biomart/martview). Then, the amino acid sequences of all known actin genes were adopted as queries in local BLASTP (Basic Local Alignment Search Tool) searches for potential homologs in seven genomes with 1e-10 as the threshold expectation value. Based on the BLASTP results, all genes were verified with the conserved actin domain by searching in corresponding Conserved Domain Database (CDD) online [28] (http://www.ncbi.nlm.nih.gov/Structure/cdd/wrpsb.cgi). Thus, the entire actin genes were identified in the seven genomes. The actin gene, in which the amino acid length of actin domain was less than 160aa, was excluded for further analysis. The associated gene name or ensembl protein id, in which this copy was not given associated gene names was used for each actin gene. The suffixal letters “Hsa,” “Ptr,” “Ggo,” “Ppy,” “Nle,” “Mmu,” and “Cja” of the gene names represented the actin copies from human, chimpanzee, gorilla, orangutan, gibbon, rhesus monkey, and marmoset genome, respectively.

### 2.2. Sequence Alignment and Phylogenetic Analysis

The amino acid sequence of actin domain was aligned by MEGA4 [29] in ClustalW with default options [30]. The resulting amino acid sequence alignments were then used to guide the alignments of nucleotide coding sequences (CDSs). Phylogenetic trees were constructed based on the bootstrap neighbor-joining method with a Jukes-Cantor model by MEGA4. The stability of internal nodes was assessed by bootstrap analysis with 1000 replicates.

**Figure 1:**
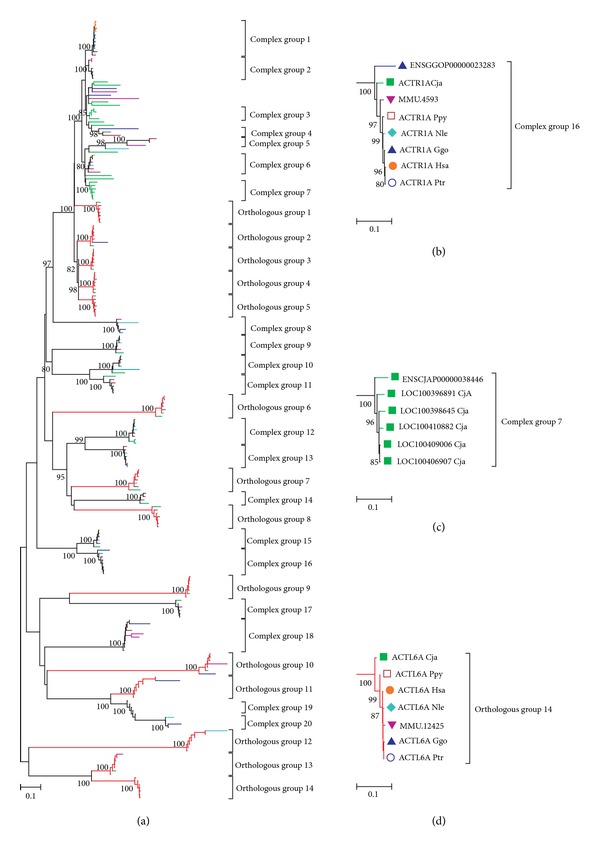
Schematic for the whole phylogenetic tree of actin genes in the seven species using nucleotide alignment of actin domain (a). *Orange* (human), *Dark Cyan* (chimpanzee), *Blue* (gorilla), *Wine* (orangutan), *Cyan* (gibbon), *Magenta* (rhesus monkey), and *Green* (marmoset). The *Red* clades represent the orthologous groups. The representative actin domain phylogenies for (b) clades in the complex group displayed multicopies from the same species, (c) clades in the complex group which lost copies from some species, and (d) clades in the orthologous group. The tree was built based on the neighboring-joining method with a Jukes-Cantor model.

Based on the nucleotide diversity/divergence between homologs within major clades (<30%) and bootstrap values, the phylogenetic tree of all actin genes from the seven genomes can be divided into two major types (Figure 1(a)) and several single genes. The first type, which contained a single copy of actin genes from each of the seven species, was designated as orthologous group, shown in Figure 1(d). The bootstrap value of every clade should be more than 80, which was considered as a credible clade. On the other hand, complex group exhibited multi-copy number or none of actin genes from one of the seven species mixed in the clade, as illustrated in Figures 1(b) and 1(c).

Nucleotide divergence among homologs was estimated by divergence (*d*) with the Jukes and Cantor correction [31]. The number of nonsynonymous substitutions per nonsynonymous site and the number of synonymous substitution per synonymous site were denoted by *K*_*a*_ and *K*_*s*_, respectively. The *K*_*a*_ and *K*_*s*_ were calculated based on Nei and Gojobori [32]. A *K*_*a*_/*K*_*s*_ ratio greater than 1 suggested positive selection, and the ratio less than 1 suggested negative selection generally. 

### 2.3. Identification of Pseudogenes

To identify actin pseudogenes, all of the nucleotide sequences of actin domains from seven species were employed to search in all the genomes used in this work (BLASTN). After excluding the hit sequences which were identified as actin genes above, a PERL script was written to remove the length of hit sequences which was shorter than 450 bp. The rest of hit positions on the chromosomes were considered as locations of actin pseudogenes. 

### 2.4. Codon Usage Estimates Using Relative Synonymous Codon Usage (RSCU)

The codon usage analysis for every actin gene was estimated by relative synonymous codon usage (RSCU) value. The RSCU value of a codon [33] is calculated by dividing the observed codon usage by that expected when all codons for the same amino acid are used equally. Due to an amino acid coded by a single codon (such as ATG: methionine and TGG: tryptophan), these two codons and stop codons were not included in an RSCU analysis. RSCU values are not affected by sequence length and amino acid frequency since these factors are eliminated during the computation. The RSCU values <1, 1, and >1 indicated that the codons used less than average, at average level (no bias), and more than average [34–36]. For actin domain nucleotide sequences of each actin gene in this study, RSCU values were calculated for the 59 relevant codons by a PERL script. The variation of RSCU value for each codon from actin genes within every genome or complex groups/orthologous groups (see Section 2.3) was calculated to estimate codon usage pattern.

### 2.5. Actin Gene Expression Analysis

The array datasets of transcription profiling of human and chimpanzee were downloaded from ARRAYEXPRESS database at the European Bioinformatics Institute (EBI, website: http://www.ebi.ac.uk/arrayexpress/). The accession number of the experiment was E-AFMX-11 [37], processing on the platform of “Affymetrix GeneChip Human Genome U133 plus 2.0 [HG-U133_Plus_2].” An R script was developed to extract the information of array probe, values of expressed level, and *P* values from the array data. The coefficient of variation (CV; SD/mean) of the expression values for actin genes was calculated to estimate expression pattern.

## 3. Result

### 3.1. Phylogeny and Classification of Actin Genes

According to the characteristic domain of actin gene (PF00022) reported previously, we identified 233 actin genes in seven genomes using BLASTP search and CDD analysis (see Supplementary Material Table S1, see Section 2.1). Based on the alignment results for actin domain sequences of all the actin genes found in the seven species, we constructed a phylogenetic tree using the Bootstrap neighbor-joining (NJ) method with a Jukes-Cantor model by MEGA v4.0 [29] (Figure 1). According to the nucleotide diversity/divergence between homologs (<30%, see Table S2) and bootstrap values (>80), we split the tree into 34 groups to investigate evolution of actin genes in detail. Under these criteria, 14 genes could not be included in any group.

The whole phylogenetic tree and representative major clades were shown in Figure S1 and Figure 1, exhibiting two dominant types of phylogenetic structures. The first type of major clades that consist of seven copies of actin genes from all species was designated as orthologous group, shown in Figure 1(d). On the other side, each clade of complex group contained more or less than one copy of actin genes from one of the seven species, as illustrated in Figures 1(b) and 1(c). For example, complex group 16 contained two copies of actin genes from gorilla genome (Figure 1(b)), while complex group 7 just included six copies from marmoset, none from other six species (Figure 1(c)).

Following the definition of two kinds of clades, there were 14 major clades (41.2% of the total clades) found in the orthologous groups, comprising 98 (42.0%) of the total actin genes. The average nucleotide divergence (*d*) of the actin domain sequences within the orthologous groups was 2.75% (Table 1). Twenty clades of complex groups were identified on the phylogenetic tree, which had 121 actin genes in the clades (Table 2). The average *d* value of the actin domain sequences in the complex groups was 5.66%, which was significantly greater than that in the orthologous groups (*P* = 0.028, using two-tailed *t*-test). In addition, the total copy number for each species in all the complex groups was from 11 to 21 (Table 2). The large *d* value and variable copy number of complex groups implied that these actin genes diverged across species.

### 3.2. Nonsynonymous to Synonymous Substitution

According to the multiple alignments of all actin genes from seven species, we calculated the average nonsynonymous substitutions (*K*_*a*_) and synonymous substitutions (*K*_*s*_) for actin domain among each pair of homologs within clades from every complex group and orthologous group (Table 1 and detail data see Table S2). Whether in orthologous groups or complex groups, the average *K*_*a*_/*K*_*s*_ ratios of most groups (82.4%) are much smaller than 1 (only six groups of average *K*_*a*_/*K*_*s*_ ratios are greater than 0.5, all of them belong to complex groups), indicating that the actin genes code highly conserved proteins because of important functions and were under strong negative selection. 

However, the average *K*_*a*_/*K*_*s*_ ratio in all the complex groups was significantly greater than that in the orthologous groups (0.346 versus 0.0941, *P* = 0.003, using two-tailed *t*-test). Furthermore, the average *K*_*a*_ in complex groups was significantly greater than that in orthologous groups (0.0404 versus 0.0106, *P* = 0.021, using two-tailed *t*-test), while the average *K*_*s*_ for both two types of clades were not significantly different from each other (0.1115 for complex groups versus 0.0842 for orthologous groups; *P* = 0.108, using two-tailed *t*-test). This suggested that actin genes included in the two types of clades experienced similar evolutionary time, but undergone uneven selections. The results confirmed that the actin genes included in the orthologous groups were higher conserved, and the actin genes from complex groups may experience a relatively relaxed negative selection during a certain period. 

At the same time, we also separately aligned the actin genes from each species and calculated the average nucleotide divergence, nonsynonymous, and synonymous substitutions in the genome (Table 1). Our results showed that the average *K*_*a*_ and *K*_*s*_ values for all pairs of paralogs in seven species were from 0.5890 to 0.8630 and from 1.2230 to 1.5680, respectively. Nevertheless, the maximum averages of *K*_*a*_ and *K*_*s*_ in both complex groups and orthologous groups were 0.2027 and 0.3317. The average *K*_*a*_ and *K*_*s*_ values for the paralogs within species were significantly greater than those for the homologous actin genes from different species within the same group (*P* < 0.001 for both *K*_*a*_ and *K*_*s*_), implying that different actin genes with distinct functions may undergo diverse selective pressures. 

### 3.3. Pseudogenes Contained Actin Domain

Discriminating pseudogene from live actin gene could help us to understand the evolutionary history of actin gene family. In our work, three-hundred and thirty-seven actin pseudogenes were identified in the seven genomes (see Section 2.3, Table S3). The number of pseudogenes was much greater than that of live actin copies. Marmoset genome has the largest number (63 copies) of pseudogenes, and the number of pseudogenes in other genomes in descending order was 59 in human, 51 in chimpanzee, 48 in orangutan, 41 in gorilla, 40 in gibbon, and 35 in rhesus monkey. All species except rhesus monkey own more dead actin genes than live ones. Actually, the number of pseudogene in rhesus monkey was equal to that of live ones, (see Table S1 and Table S3). The frameshift insertions or deletions and premature stop codons were observed in all the pseudogene sequences. The dead actin genes abundantly existing in all the seven genomes provided evidence that actin genes went through a duplication first and then varied in the evolutionary process.

### 3.4. Codon Usage

**Figure 2:**
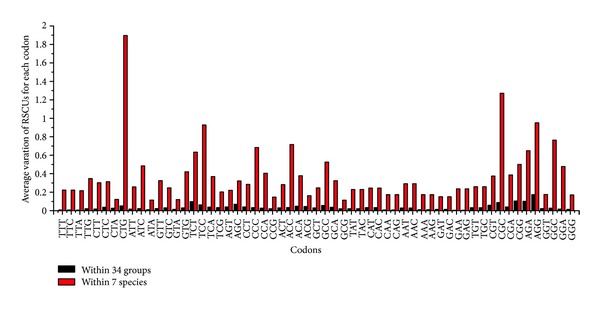
Variations of relative synonymous codon usage (RSCU) values for actin genes within groups (black columns) and species (red columns) in each codon. Each column represents average variations of all groups (or species) for one codon. For all the 59 codons, the red columns are significantly higher than black ones (*P* < 0.001 for all codons, detailed corresponding *P* value for each codon see Table S4 in Supplementary Material available online at http://dx.doi.org/10.1155/2013/630803), representing that the codon usage patterns of actin genes within species were very distinct among each other, while those of actin genes within groups were much similar.

The synonymous codons, which code for the same amino acid, were reported to be used unequally in almost all species [38–43] and present the evolutionary pattern of genes. For this reason, the study of codon usage pattern could be helpful to understand actin gene family. To study the codon usage of actin genes within species and within groups (including complex groups and orthologous groups split from the tree), all the actin domain sequences of copies were examined by RSCU values of the 59 relevant codons (see Section 2.4). The variation of RSCU values for each codon from actin genes within each group and every species was calculated to examine the extent of difference in codon usage pattern. The larger the variation value was, the more various codon usage patterns among the groups or species there were. The differences of average variations were revealed between 34 groups and 7 species for every codon (Figure 2). The average variations in all the species were significantly greater than those in 34 groups for the total 59 codons (*P* < 0.001 for all codons, using two-tailed *t*-test, see Table S4). These results demonstrated that actin genes within groups come from different species that had relative coincident codon usage pattern, while the codon usage of actin genes within species diverged a lot. 

### 3.5. Actin Gene Expression Pattern

**Figure 3:**
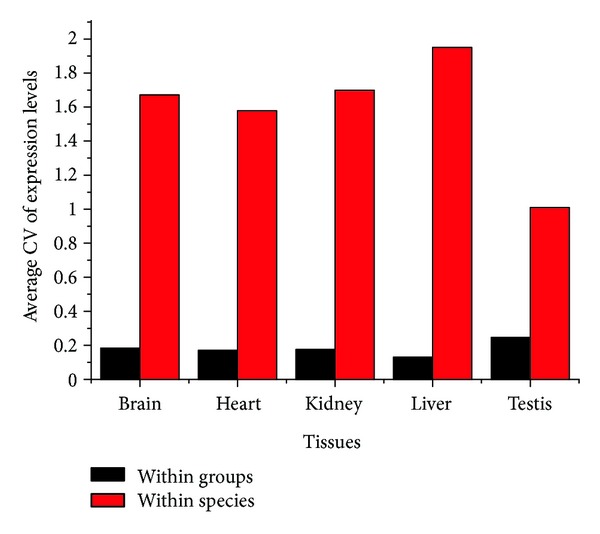
Coefficient of variations (CVs) of gene expression values for actin genes within groups (black columns) and species (red columns) in each tissue. Each column represents average CVs of all groups (or species) for one tissue. For all the five tissues, the black columns are significantly lower than red ones, indicating that the actin genes within groups exhibited similar expression patterns, while actin genes within species did not (*P* < 0.001, using two-tailed *t*-test).

The transcription profiling data of humans and chimpanzees in brain, heart, liver, kidney, and testis were employed to detect whether there were any differences of the expression patterns for actin genes between and within species [37]. The expression level values of 23 actin genes from human and 21 from chimpanzee in the five tissues were extracted from array data. Because the gene expression data were measured in multiple samples, with the addition of some actin genes represented by more than one corresponding probes, the average value of actin gene expression using every probe in all samples was adopted as the expression level for this actin copy in each tissue. The extent of difference in gene expression between paralogs and orthologs was measured by coefficient of variation (CV) of expression values (Table S5). In our results, a large proportion of the CV's (65/67) for actin genes within groups in the five tissues was smaller than 0.4; however, the minimum CV of actin genes within species was 0.875, and 80% of them were greater than 1.50 as well. The significant differences of average CV results between paralogs and orthologs in all the tissues were shown as in Figure 3 (*P* < 0.001, using two-tailed *t*-test). These results demonstrated that actin genes within species, which possessed distinct categories of functions, had differential expression levels among each other, whereas actin genes within groups but from different species, which might be involved in the identical function, expressed in the same level.

## 4. Discussion

### 4.1. Phylogenetic Analysis

Actin is reported as an abundant cytoskeletal protein that plays a central role in many cellular processes. The phylogenetic analysis of actin genes in multicellular animals showed that phylogeny corresponded well with distinct functional categories into, for example, cytosolic, smooth, and cardiac muscle actins [44] and more divergent actin-related proteins [13, 14]. However, the phylogenetic structures in dinoflagellates exhibited at least three types of clusters [26]. 

Based on our results, the apparent feature of orthologous groups was one actin gene copy from each species clustered together on the phylogenetic tree, possessing distinct functions, which were coincident with Oota's and Muller's results [13, 44]. Nevertheless, more than 50% of the total actin genes incompletely interspecifically or monophyletically clustered on the clades formed complex groups. In fact, actin genes within the complex groups could be divided into three types in detail, based on the branch length and organization of the groups. The first type consisted of complex groups 1, 3, 7, 12, and 13, which had more than one copy from a species in the clades, indicating recent duplication that occurred after speciation. The complex groups 4 and 5, which contained much more divergent actin gene sequences than the other groups did (see Table 1), were designated as type 2. These actin genes would possibly subject to faster relative mutation rate or longer divergence time than other genes. And the other complex groups belong to the third type, in which one or two orthologous copies were lost in some primates. Furthermore, 85% of the lost copies in the third type were found to become pseudogenes in the corresponding genomes or have truncated actin domain which were excluded in the work (the nucleotide length of actin domain was smaller than 160 bp). Thus, actin gene sequences within the three types of complex groups plus the orthologous groups in the primates, which had similar phylogenetic structures in dinoflagellates at some extent [26], appeared to have diverged from one another at different time points during and after speciation. The copy number variation on the phylogenetic tree reflected complicated evolutionary patterns of actin gene family. The results also implied that the actin gene family might obtain new function or alter original function by changing copy number in the genome during the evolutionary process.

### 4.2. Distinct Selection

Actin genes within orthologous groups and complex groups showed significantly different levels of nucleotide diversity, *K*_*a*_ and *K*_*a*_/*K*_*s*_ ratios, suggesting they had undergone nonuniformly selections. The *K*_*a*_/*K*_*s*_ ratios in the orthologous groups were significantly smaller than those in the complex groups (Table 1), implying that actin genes within orthologous groups were highly conserved under strong background selection owning to their basic functions in the cells. On the other hand, relaxation of negative selection or positive selection was associated with copy number variation, which was detected in complex groups, leading to relatively rapid diversification of actin genes within complex groups.

At the same time, we found that actin genes were tremendously divergent from each other within species, which the average nucleotide diversity, *K*_*a*_ and *K*_*s*_ of the actin domain sequences in each genome was greater than 0.70, 0.58 and 1.20, respectively (Table 1). This result was much unexpected, for actin was one of the most highly conserved proteins [24]. Actins in plant genomes, such as *Populus* and *Arabidopsis thaliana*, were reported to share high sequence homology, larger than 90% identity [45, 46]. Similarly, *K*_*a*_ and *K*_*s*_ for actin genes in dinoflagellate species were less than 0.05 and 0.55 [26], much smaller than our results. In consideration of the fundamental importance of actins, we inferred that molecular diversification of actin gene family could result in functional diversification in the complex higher organisms like primates. Besides, 82.8% of actins were conserved across species instead of within species, suggesting that parallel selection played a major role in the evolution of actins.

### 4.3. The Different Characteristics of Actin Genes across and within Species

On the basis of the transcription profiling data of humans and chimpanzee in brain, heart, liver, kidney, and testis, 72.7% of actin genes appear to be differentially expressed in different tissues. The actin genes, twelve from human and ten from chimpanzee, have available array data in all the five tissues (see Table S6), for which the average CVs of expression level values were 0.584 and 0.527 of human and chimpanzee, respectively. The CVs for 16 out of 22 actin genes were greater than 0.4, significantly greater than those for almost all the orthologs of actin genes between species, suggesting overlapping and unique expression patterns of actin gene family members due to distinct functions. The findings were in agreement with previous studies, in which the isoforms of nonplant actin appear to be differentially expressed in striated muscle, smooth muscle, and nonmuscle tissues [47], and individual actins from plants, such as *Arabidopsis thaliana* and *Populus*, show specific expression patterns, congruent with their evolutionary relationships [45, 46, 48–50].

In addition, the average CVs of expressed values for actin copies within species in every tissue were significantly greater than those of each actin copy among the tissues, implying that actin genes with distinct functions had different expression levels. The CVs of gene expression values for actin copes within species were much greater than those for actin genes within groups across species, suggesting a big difference between paralogs and orthologs of actin copies. Besides, similar results in the codon usage pattern as well as the findings for divergence and ratio of nonsynonymous to synonymous substitutions were also revealed between paralogs and orthologs of actin copies, implying that actin copies were highly homologous within groups. All these results might provide a clue for paralogs and orthologs of actin copies through different evolutionary histories.

### 4.4. Dynamic Actin Gene Family Evolution in Primates

In agreement with previous studies, actin was highly conserved across primates due to its important functions, proved by our results that about 40% of actin genes belong to the orthologous groups with well-interspecific distribution and little divergence. On the other hand, actin was needed to obtain new function constantly in order to adapt more and more complicated system in complex higher organisms. How did actin evolve to meet the pair of conflict demands in primates?

Generally speaking, gene acquired new function resulting from increase of self-complexity or copy number variation. Increasing gene length or fusing with other domains could increase its complex, while duplication offered a chance to gain new functions without losing the original ones. 

Interestingly, several actins were found to fuse with other domains to generate new functions. For example, actins within complex group 1 also contained POTE ankyrin domain [51, 52]. And a length of DUF1542 domain sequences was examined to insert into actin genes within complex group 17 that encode *ACTR5* protein. Similarly, actin domain of *ACTR8* genes comprising orthologous group 12 was encompassed in COG5277 domain. However, the rest of the actin genes comprising complex groups chose the other way. The tremendous pseudogenes, presenting for the copies that failed to gain new function, also gave an evidence for the numerous duplications of actin genes. The organization of actins and characteristics of actin gene family indicated that actins acquired new function in various evolutionary pathways. Both of increasing self-complexity and copy number, especially the second way, played important roles in promoting the evolution of actin.

Taken together, several evolutionary characteristics of actin gene family in primates were observed in our results. First, the phylogenetic tree structure for all the actin domain sequences exhibited that 89.7% of actin genes clustered with other orthologous copies from distinct species, implying incomplete lineage sorting [53] during the divergence of the seven primates and inconsistent divergence time or rate of variance between gene copies. Second, the differences of divergences, codon usage, and expression patterns between orthologs and paralogs of actin copies within groups and within species indicated that actin genes within groups were highly homologous, but actin genes within species were very divergent. Therefore, we deduced that multiple rounds of gene duplication events have occurred and that the most multiple actin gene homologs likely existed in the recent common ancestor. Finally, the presence of a great deal of pseudogenes provided convinced evidence for actin gene experiencing duplicated, mutated, and dead process. We conclude that gene family expansion and contraction have continued during and after speciation of these primates. These features of actin gene family in primates provided evidence for us to explain how actin gene family evolved leading to the contradictory characteristics of conserved across species but divergent within species in the evolutionary history in primates. 

Generally, “concerted evolution” and “birth and death” models were often invoked to explain the divergence and evolution of multigene families [54]. Under concerted evolution model, new gene copies were duplicated, homogenized, and deleted by interlocus recombination or intergenic gene conversion, resulting in a high degree of sequence similarity among multigene family members [55–57]. In contrast, under the model of “birth and death,” new gene copy was created by various forms of gene duplications in order to diverge functionally, in which some duplicated copies with new function or original function maintained in the genomes, and others became nonfunctional or deleted due to mutation and degeneration. Thus, the predicted end result of this model was a mixture of divergent groups of genes and highly homologous genes within groups plus many great pseudogenes present in the multigene family [54]. 

Obviously, although actin gene copies from the same species shared highly similar sequences clustered on the first type of complex groups partly corresponded with convert evolution model, the major characteristics of actin genes, such as the variation in copy number, the structure of the phylogenetic tree with a mixture of divergent groups of gene copies, the differences of divergences, codon usage, expression patterns between orthologs and paralogs of actin genes across and within species, and the presence of many pseudogenes, fit well with “birth and death” model of multigene family evolution [54]. 

Since actin family plays such a crucial role in all aspects of cell activities, their related functions cannot be easily altered or removed. However, the way of the copy number of actin genes changed following “birth and death model” maybe affording an alternative evolutionary pathway to meet the conflicting demands that actin was conserved to maintain vital functions and evolved new functions in the body in order to help adapting to environmental pressure. Under this scenario, organisms may not only keep bodies working regularly, but make species evolving from simple to complex, from rough to fine. We infer that birth and death evolution model might be a common evolutionary mechanism in other highly conserved multigene families.

## 5. Conclusions

In summary, 233 actin genes and 337 pseudogenes were identified in the seven primates. Phylogenetic analysis for actin genes exhibited two major types of clades. Actin genes interspecifically clustered that belong to the orthologous groups were highly conserved because of fundamental importance. On the contrary, complex groups contained actin gene members that displayed copy number variation with significantly higher levels of average nucleotide divergence and *K*_*a*_/*K*_*s*_ ratios compared to the orthologous groups. Analysis of codon bias and gene expression level revealed that actin genes in primates were extraordinarily divergent from each other within species, but were highly conserved within groups across species. These results may be explained by a birth and death evolutionary process of actin gene families, which would be the general evolutionary mechanism for other highly conserved multigene families. 

## Supplementary Material

Supplementary Figure: The whole phylogenetic tree of actin genes in the seven species using nucleotide alignment of actin domain. The tree was built based on the Neighboring-joining method with a Jukes-cantor model.
Supplementary Table 1: The number of actin genes with their CDS length in seven genomes.
Supplementary Table 2: Nucleotide diversity, nonsynonymous and synonymous substitutions of each pair of orthologs or paralogs of actin genes for actin domain within every group.
Supplementary Table 3: Information of location and strand on the chromosome for each pseudogene identified in the seven species.
Supplementary Table 4: List of the corresponding P value for the two tailed t-tests used to determinate if the average variation was significant different between 34 groups and 7 species for each codon.
Supplementary Table 5: The coefficient of variation of the expression level values for actin genes within species and within different groups in all the five tissues.
Supplementary Table 6: Expression level of actin genes from human and chimpanzee genomes in all the five tissues.

## Figures and Tables

**Table 1:** Average nucleotide diversity, nonsynonymous, and synonymous substitutions of actin domain from actin genes in each group and species.

Name	*d*	*K*_*a*_	*K*_*s*_	*K*_*a*_/*K*_*s*_
Orthologous group 1	0.0234	0.0058	0.0819	0.0711
Orthologous group 2	0.0374	0.0188	0.1002	0.1874
Orthologous group 3	0.0149	0.0000	0.0642	0.0000
Orthologous group 4	0.0163	0.0010	0.0674	0.0153
Orthologous group 5	0.0204	0.0000	0.0902	0.0000
Orthologous group 6	0.0362	0.0156	0.1050	0.1488
Orthologous group 7	0.0510	0.0262	0.1268	0.2064
Orthologous group 8	0.0366	0.0184	0.0899	0.2047
Orthologous group 9	0.0107	0.0005	0.0483	0.0107
Orthologous group 10	0.0777	0.0606	0.1349	0.4487
Orthologous group 11	0.0094	0.0000	0.0416	0.0000
Orthologous group 12	0.0186	0.0006	0.0831	0.0069
Orthologous group 13	0.0207	0.0007	0.0893	0.0077
Orthologous group 14	0.0125	0.0005	0.0563	0.0089
Average for orthologous groups	**0.0276**	**0.0106**	**0.0842**	**0.0940**
Complex group 1	0.0208	0.0136	0.0445	0.3067
Complex group 2	0.0183	0.0011	0.0755	0.0146
Complex group 3	0.0350	0.0364	0.0305	1.1935
Complex group 4	0.2291	0.2027	0.3317	0.6112
Complex group 5	0.1455	0.1362	0.1770	0.7697
Complex group 6	0.0494	0.0183	0.1570	0.1164
Complex group 7	0.0364	0.0236	0.0793	0.2973
Complex group 8	0.0760	0.0674	0.1053	0.6399
Complex group 9	0.0324	0.0199	0.0754	0.2647
Complex group 11	0.0555	0.0312	0.1382	0.2255
Complex group 10	0.0945	0.0735	0.1574	0.4668
Complex group 12	0.0392	0.0123	0.1343	0.0919
Complex group 13	0.0217	0.0081	0.0657	0.1229
Complex group 14	0.0516	0.0376	0.0928	0.4054
Complex group 15	0.0330	0.0022	0.1388	0.0155
Complex group 16	0.0380	0.0158	0.1133	0.1397
Complex group 17	0.0265	0.0068	0.0924	0.0736
Complex group 18	0.0630	0.0540	0.0931	0.5797
Complex group 19	0.0107	0.0000	0.0466	0.0000
Complex group 20	0.0554	0.0482	0.0816	0.5909
Average for complex groups	**0.0566**	**0.0404**	**0.1115 **	**0.3463**
Homo sapiens (human)	0.8350	0.6650	1.3310	0.4996
Callithrix jacchus (marmoset)	0.7360	0.5890	1.2230	0.4816
Gorilla gorilla (gorilla)	0.8510	0.6890	1.3410	0.5138
Macaca mulatta (rhesus monkey)	0.9040	0.7270	1.4500	0.5014
Nomascus leucogenys (Gibbon)	1.0050	0.8630	1.5680	0.5504
*Pan troglodytes* (chimpanzee)	0.9980	0.8300	1.4780	0.5616
*Pongo pygmaeus abelii* (orangutan)	0.8830	0.7050	1.5140	0.4657
Average for all species	**0.8874 **	**0.7240 **	**1.4150 **	**0.5106 **

**Table 2:** Distribution of actin copies from seven species in each complex group.

Group name	Human	Chimpanzee	Gorilla	Orangutan	Gibbon	Rhesus monkey	Marmoset	Total
Complex group 1	3	4	2	1	0	0	0	10
Complex group 2	2	1	1	1	1	2	0	8
Complex group 3	0	0	0	0	0	0	4	4
Complex group 4	0	1	1	1	0	0	0	3
Complex group 5	0	1	1	0	0	1	0	3
Complex group 6	1	0	1	1	1	2	0	6
Complex group 7	0	0	0	0	0	0	6	6
Complex group 8	0	1	1	1	1	1	0	5
Complex group 9	1	1	1	0	1	1	1	6
Complex group 11	1	1	1	1	0	1	1	6
Complex group 10	1	1	1	2	0	0	1	6
Complex group 12	1	1	1	1	1	1	2	8
Complex group 13	1	1	2	1	1	1	0	7
Complex group 14	0	0	1	1	0	1	1	4
Complex group 15	1	1	0	1	1	1	1	6
Complex group 16	1	1	2	1	1	1	1	8
Complex group 17	1	1	0	1	1	1	1	6
Complex group 18	1	1	1	2	1	3	1	10
Complex group 19	1	1	0	1	0	1	1	5
Complex group 20	0	2	0	0	1	1	0	4

Total	16	20	17	17	11	19	21	121

## Abbreviations

CDS: Coding sequences
*d*: Divergence/diversity
*K*_*a*_: Nonsynonymous substitutions
*K*_*s*_: Synonymous substitutions
RSCU: Relative synonymous codon usage
CV: Coefficient of variation
CDD: Conserved domain database
BLAST: Basic Local Alignment Search Tool.
