# Supplementary figures and images for: Dynamic Actin Gene Family Evolution in Primates
Source: Biomed Res Int. 2013 Jun 6;2013:630803. doi: 10.1155/2013/630803 (PMC3690210; doi:10.1155/2013/630803)

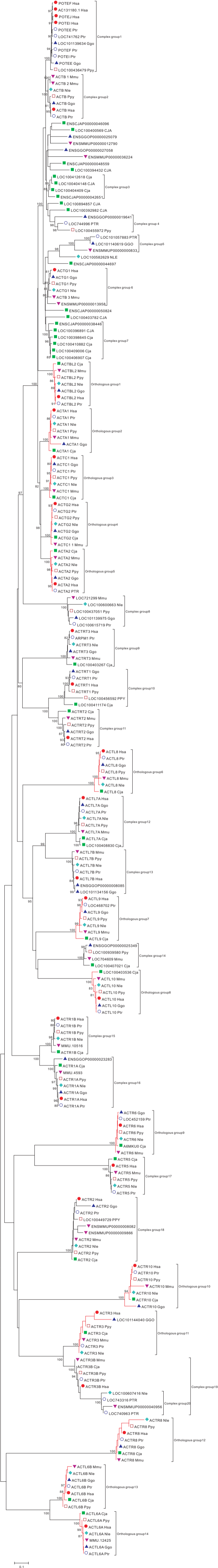

Supplement: Supplementary file 1 — Supplementary Figure: The whole phylogenetic tree of actin genes in the seven species using nucleotide alignment of actin domain. The tree was built based on the Neighboring-joining method with a Jukes-cantor model. Supplementary Table 1: The number of actin genes with their CDS length in seven genomes. Supplementary Table 2: Nucleotide diversity, nonsynonymous and synonymous substitutions of each pair of orthologs or paralogs of actin genes for actin domain within every group. Supplementary Table 3: Information of location and strand on the chromosome for each pseudogene identified in the seven species. Supplementary Table 4: List of the corresponding P value for the two tailed t-tests used to determinate if the average variation was significant different between 34 groups and 7 species for each codon. Supplementary Table 5: The coefficient of variation of the expression level values for actin genes within species and within different groups in all the five tissues. Supplementary Table 6: Expression level of actin genes from human and chimpanzee genomes in all the five tissues. [file 630803.f1.pdf]
